# An unconventional RNA-based thermosensor within the 5’ UTR of *Staphylococcus aureus cidA*

**DOI:** 10.1371/journal.pone.0214521

**Published:** 2019-04-01

**Authors:** Hebaallaha Hussein, Megan E. Fris, Ahmed H. Salem, Richard E. Wiemels, Raeven A. Bastock, Francesco Righetti, Caleb A. Burke, Franz Narberhaus, Ronan K. Carroll, Nahla S. Hassan, Saleh A. Mohamed, Afaf S. Fahmy, Erin R. Murphy

**Affiliations:** 1 National Research Centre, Cairo, Egypt; 2 Ohio University, Department of Biological Sciences, Athens, OH, United States of America; 3 Infection and Topical Disease Institute, Ohio University, Athens, OH, United States of America; 4 Ain Shams University, Cairo, Egypt; 5 Molecular and Cellular Biology Program, Ohio University, Athens, OH, United States of America; 6 Ruhr-Universität Bochum, Bochum, Germany; 7 Ohio University Heritage College of Osteopathic Medicine, Department of Biomedical Sciences, Athens, OH, United States of America; Hosei University, JAPAN

## Abstract

*Staphylococcus aureus* is a Gram-positive bacterial pathogen of global concern and a leading cause of bacterial infections worldwide. Asymptomatic carriage of *S*. *aureus* on the skin and in the anterior nares is common and recognized as a predisposing factor to invasive infection. Transition of *S*. *aureus* from the carriage state to that of invasive infection is often accompanied by a temperature upshift from approximately 33°C to 37°C. Such a temperature shift is known in other pathogens to influence gene expression, often resulting in increased production of factors that promote survival or virulence within the host. One mechanism by which bacteria modulate gene expression in response to temperature is by the regulatory activity of RNA-based thermosensors, *cis-*acting riboregulators that control translation efficiency. This study was designed to identify and characterize RNA-based thermosensors in *S*. *aureus*. Initially predicted by *in silico* analyses of the *S*. *aureus* USA300 genome, reporter-based gene expression analyses and site-specific mutagenesis were performed to demonstrate the presence of a functional thermosensor within the 5’ UTR of *cidA*, a gene implicated in biofilm formation and survival of the pathogen. The nucleic sequence composing the identified thermosensor are sufficient to confer temperature-dependent post-transcriptional regulation, and activity is predictably altered by the introduction of site-specific mutations designed to stabilize or destabilize the structure within the identified thermosensor. The identified regulator is functional in both the native bacterial host *S*. *aureus* and in the distally related species *Escherichia coli*, suggesting that its regulatory activity is independent of host-specific factors. Interestingly, unlike the majority of bacterial RNA-based thermosensors characterized to date, the *cidA* thermosensor facilitates increased target gene expression at lower temperatures. In addition to the characterization of the first RNA-based thermosensor in the significant pathogen *S*. *aureus*, it highlights the diversity of function within this important class of ribo-regulators.

## Introduction

*Staphylococcus aureus* is a Gram-positive pathogen that causes both nosocomial and community-acquired infections, ranging in severity from food poisoning and skin abscesses, to life-threatening diseases such as pneumonia, meningitis, endocarditis, necrotizing fasciitis, and toxic shock syndrome [1, 2]. Before antibiotics were available, mortality associated with *S*. *aureus* infections approached 80% [3]. *S*. *aureus* infections remain difficult to treat despite modern antibiotics due to the ability of the bacteria to persist and adapt to the surrounding environment, as well as the ever-increasing rate of antibiotic resistance seen within the species [4–7]. In contrast to the clinical presentation of severe invasive disease, *S*. *aureus* can be carried asymptomatically on the skin and in the anterior nares of the human host. Such carriage is common and considered a significant predisposing factor to invasive disease [8].

Bacterial survival is dependent upon the ability to sense and rapidly adapt to specific changes within the immediate environment. One variable environmental condition known to influence bacterial gene expression is temperature. While extreme alterations in temperature can be deleterious to bacterial survival, more subtle changes in environmental temperature, such as that experienced by *S*. *aureus* during the transition from carriage to invasive disease, can represent an important environmental cue. When experienced by pathogenic bacteria, subtle shifts in environmental temperature often influence virulence gene expression [9–11].

One mechanism by which bacteria sense environmental temperature is via cis-acting RNA-based thermosensors; RNA elements with temperature-responsive secondary structure [12]. Particularly significant are temperature induced changes within mRNA transcripts that alter the structure of the region composing and surrounding the ribosomal binding site. The majority of RNA-based thermosensors characterized to date are RNA thermometers (RNATs), a class of regulators first identified in bacteriophage lambda [12, 13]. Since that time, RNATs have been identified in a variety of bacterial species and implicated in the thermo-regulation of factors involved in central functions ranging from heat-shock response, to nutrient acquisition and other virulence-associated processes [11, 12, 14]. With the exception of a single RNAT in *Listeria monocytogenes* [15], RNA-based thermosensors have been investigated exclusively in Gram-negative genera including *Shigella*, *Salmonella*, *Escherichia*, *Vibrio*, *Neisseria*, *Pseudomonas* and *Yersinia* [11, 16–23]. Despite its recognition as a significant human pathogen, and its widely acknowledged ability to transition from a state of carriage to that of invasive disease (a process associated with a change in environmental temperature), no RNA-based thermosensors have been predicted, identified, or experimentally characterized in *S*. *aureus*.

The studies presented herein are the first to predict and experimentally investigate the existence of functional RNA-based thermosensors in *S*. *aureus*. Initially predicted by *in silico* analysis using the genome of the community associated methicillin resistant *S*. *aureus* (CA-MRSA) strain USA300, subsequent *in vitro* and *in vivo* analyses demonstrate the presence of a functional RNA-based thermosensor in the 5’ untranslated region (UTR) of the *cidA* gene [24, 25]. Importantly, these data indicate that the identified *cidA* thermosensor functions to facilitate efficient translation at relatively low temperature; a feature that differentiates this ribo-regulator from typical bacterial RNATs that function to facilitate translation at relatively high temperatures. Thus, these are the first studies to identify a functional RNA-based thermosensor in *S*. *aureus* and highlight the diversity among this important family of ribo-regulators.

## Materials and methods

### Strains, plasmids and growth conditions

All strains and plasmid used in this study are detailed in Table 1.

**Table 1:** Strains and plasmids.

**Strains**	**Description**	**Source**
*E*. *coli* DH5α	Containing the reporter constructs	Life Technologies
*S*. *aureus* USA300 TCH1516	Community associated USA300 MRSA isolate	[26]
*S*. *aureus* RN4220	Restriction deficient transformation recipient strain	[27]
*S*. *aureus* RN9011	RN4220 pRN7023	[28]
*S*. *aureus* SAPI1::*cidA*-*gfp*	RN9011 containing the *cidA*-*gfp* construct inserted into the SAPI1 transposon site	This study
**Plasmid**	**Description**	**Source**
pXG-10	Plasmid containing a constitutive promoter and *gfp* reporter gene	[29]
p*sspB*-UTR	Translational reporter plasmid containing the putative *sspB* thermoregulator	This study
p*ssaA*-UTR	Translational reporter plasmid containing the putative *ssaA* thermoregulator	This study
pT-*cidA*-UTR	Translational reporter plasmid containing the 36 nt long *cidA* 5’ UTR	This study
p*cidA*-UTR	Translational reporter plasmid containing the 82 nt long *cidA* 5’ UTR	This study
pS-*cidA*-UTR	Translational reporter plasmid containing point mutations predicted to stabilize the *cidA* thermoregulator	This study
pD-*cidA*-UTR	Translational reporter plasmid containing point mutations predicted to destabilize the *cidA* thermoregulator	This study
pJC1112	Integration vector	[28]
pJC1112-prom*-cidA*-*gfp*	pJC1112 containing the native *S*. *aureus cidA* promoter and 5’ UTR cloned in frame with a reporter *gfp*	This study

All *E*. *coli* strains were cultured on Luria-Bertani (LB) agar or in LB broth at the indicated temperature. When indicated chloramphenicol was used at 30μg/ml to select for, and ensure maintenance of, a given translational reporter plasmid. All *S*. *aureus* strains were cultured on TSB agar or in tryptic soy broth (TBS) broth at the indicated temperature. When indicated ampicillin was used at 100μg/ml and erythromycin was used at 5μg/ml for the selection of the replicating or indicated reporter plasmid, respectively.

### *In silico* prediction of RNA-based thermosensors in *S*. *aureus*

In order to identify potential RNA-based thermosensors in the *S*. *aureus* genome, a script was developed using Python (v 2.7.4). The genome sequence and the coding sequence FASTA files of *S*. *aureus* USA300 (CP000255.1) were downloaded from the NCBI repository and used as input. The script extracts a list of all the annotated genes and the hypothetical 5´ UTRs defined as the regions between –70 and +30 nt from the translational start codon. These sequences were then folded *in silico* with RNAfold (Vienna RNA Package v 2.3.0) with default parameters (37°C) [30]. A filtering procedure was used to extract those 5’ UTRs in which at least 6 of the nt within the ribosome binding region, defined by the user as the locus between -20 and -6 from the start codon position, are predicted by RNAfold to be base-paired. The final output contains a list of 5´UTRs containing putative RNA-based thermosensors and corresponding structures. The output list contained a total of 277 candidate *S*. *aureus* thermosensors.

### Generation of wild-type and mutated translational reporter plasmids

Reporter plasmids were generated by the following procedure. Complementary DNA oligonucleotides composing the nucleic acid sequence of the wild-type or mutated *cidA* 5’ UTRs along with NheI and NsiI overhangs were custom synthesized by Integrated DNA Technologies (Skokie, IL). Complementary oligos were resuspended to 100pm/*μ*l in 1X STE buffer and annealed by boiling in a water bath for 10 minutes followed by slow cooling to room temperature. Annealed oligonucleotides were subsequently ligated into plasmid pXG-10 [29] previously digested with *Nhe*I and *Nsi*I, placing the thermosensor between the constitutive pLtetO-1 plasmid promoter and the ATG-less *gfp* reporter gene. After ligation, reporter plasmids were introduced into chemically competent *E*. *coli* DH5α by heat shock transformation and colonies carrying each reporter plasmid were selected for by growth on Luria-Bertani (LB) agar plates containing 30 μg/ml chloramphenicol. All reporter plasmids were sequence verified by Sanger sequencing in the forward and reverse direction (Ohio University Genomics Facility, Athens, OH).

### Western blot analyses

Western blot analyses were performed on whole cell extracts. Bacteria containing each thermosensor-*gfp* reporter plasmid were cultured to mid-logarithmic growth phase at the indicated temperatures in a shaking incubator. Next, 5x10^8^ cells were pelleted by centrifugation at 17,000xg for 2 minutes and the pellet was resuspended in 100μl of Laemmli protein dye (Bio-Rad, Hercules, CA) containing 5% β-mercaptoethanol. Samples were then boiled for 10 minutes and stored at -20°C until use. 15μl of each protein sample was loaded onto a 15% polyacrylamide gel and separated using sodium dodecyl sulfate polyacrylamide gel electrophoresis (SDS-PAGE) at 80V for 20 minutes followed by 120V for 120 minutes. A polyvinylidene diflouride (PVDF) membrane cut to the size of the gel was prepared by soaking in methanol for 5 minutes, rinsing with water for 2 minutes and then equilibrated in transfer buffer for 1 minute. Protein was then transferred from the acrylamide gel to the prepared PVDF membrane at 350 milliamperes for 60 minutes. Each membrane was then incubated overnight at 4°C in blocking solution (PBS containing 0.1% (vol/vol) Tween 20 (PBST) and 10% (wt/vol) non-fat milk). The following day, each membrane was incubated for 1 hour in primary antibody (mouse anti-GFP (Roche, Basel, Switzerland) diluted 1:1000 in PBST and 5% (wt/vol) non-fat milk). Each membrane was then washed 3 times in PBST for 5 minutes each followed by incubation for 1 hour at 4°C in secondary antibody (goat anti-mouse HRP conjugated IgG (Bio-Rad, Hercules, CA) diluted 1:20,000 in PBST and 5% (wt/vol) non-fat milk). Each membrane was subsequently washed three times in PBST for 15 minutes each prior to incubation for 3 minutes in chemiluminescent HRP substrate as per instruction (Millipore, Billerica MA). Signal was visualized, and the relative intensity calculated using a Molecular Imager ChemiDoc XRS+ imaging system (Bio-Rad, Hercules, CA).

### RNA isolation

*E*. *coli*: DH5α containing a given reporter plasmid was grown in LB broth with 30μg/ml chloramphenicol in a shaking incubator at the indicated temperature until the mid-logarithmic growth phase of growth (OD_600_≅0.4). Following the removal of an aliquot to be used for Western Blot analysis (see above), 250μl of RNA-stay (95% EtOH and 5% phenol) per ml was added to each culture and the sample was incubated overnight at 4°C. Bacteria present in each sample were pelleted by centrifugation in a microcentrifuge at 17,000xg for 2 minutes. Bacterial pellets were resuspended in 357.3μl of RNase free H_2_O, 40μl of 10% sodium dodecyl sulfate (SDS), and 2.67μl 3M sodium acetate (pH 5.2). Samples were vortexed for 15 seconds and subsequently heated to 90°C for 7 minutes. Next, 1ml of TRIzol (Thermo Fisher Scientific, Waltham, MA) was added to each sample and mixed by pipetting. Samples were transferred to a 5PRIME phase lock tube (Quantabio, Beverly, MA) and incubated at room temperature for 5 minutes. Following this incubation, 250μl of chloroform was added to each tube and the tube shaken vigorously for approximately 1 minute. Samples were then incubated for 2 minutes at room temperature followed by centrifugation at 17,000xg for 2 minutes. The upper phase of each sample was transferred to a new microcentrifuge tube containing 1ml ice cold 100% EtOH, inverted twice, and incubated overnight at -80°C. The following day, samples were centrifuged at 17,000xg for 15 minutes at 4°C, the supernatant removed, and the pellet washed with 1ml ice cold 75% EtOH by centrifugation as above. The final supernatant was removed, and the RNA pellets were dried in a micro vacufuge. Final RNA pellets were resuspended in 54μl of RNAase free H_2_O. All RNA samples were treated with the Turbo DNA Free Kit (Ambion, Austin, TX) according to the manufacturer’s instructions and screened for DNA contamination using a screening PCR designed to detect *sodB*. Finally, RNA was measured for quantity and purity using a Nanodrop 2000c spectrophotometer (Thermo Fisher Scientific, Waltham, MA).

*S*. *aureus*: RN4220 containing the SAP1::*cidA*-*gfp* reporter was grown in TSB to the mid-logarithmic growth phase (OD_600_≅0.6) at the indicated temperatures in a shaking incubator. RNA was isolated from *S*. *aureus* using the RNeasy kit (QIAGEN, Hilden, Germany) with modifications [31]. RNA samples were treated with the Turbo DNA Free Kit (Ambion, Austin, TX) according to the manufacturer’s instructions. Finally, the quantity and purity of each RNA sample was measured using a Nanodrop 2000c spectrophotometer (Thermo Fisher Scientific, Waltham, MA). RNA integrity was evaluated by running 5μl of each RNA sample on a 1% agarose gel stained with ethidium bromide.

### cDNA generation

After the isolation of total RNA and subsequent DNA removal as detailed above, cDNA was generated using the iScript cDNA Synthesis Kit (Bio-Rad, Hercules, CA) according to the manufacturer’s protocol.

### Quantitative real-time PCR analysis

Five μl of diluted cDNA was combined with 10μl of iTaq Universal SYBR Green Supermix (Bio-Rad, Hercules, CA) and 5μl of each primer set at a validated optimum concentration. Amplification and detection were performed in a CFX96 Real-Time System (Bio-Rad, Hercules, CA) under conditions optimized for each gene target. For each gene target on each reaction plate, a six-point standard curve was included in order to ensure acceptable amplification efficiency and that all experimental samples fall within the linear range portion of the standard curve. The relative amount of each target transcript was calculated using the ΔΔCt method with experimental Ct values normalized to that of *rrsA* (*E*. *coli*) or *gyrB* (*S*. *aureus*) present in each sample and expressed relative to the levels of the target gene in a single selected control sample.

### Generation of the integration reporter strain

An integration plasmid was generated using a SOEing PCR reaction. The native *cidA* promoter and upstream sequences were amplified from *S*. *aureus* genomic DNA. Next, the *cidA* 5’ UTR and *gfp* gene were amplified from p*cidA*-UTR. The resulting amplicons were combined, and a secondary PCR was performed with the outermost primers resulting in a combined amplicon containing upstream sequences, the *cidA* promoter, the 5’ UTR of *cidA* and the *gfp* gene in frame. The large amplicon was subsequently ligated into the pZero-Blunt TOPO plasmid (Thermo Fisher Scientific, Waltham, MA). The resulting plasmid was digested with *Eco*RI (New England Biolabs, Ipswitch, MA) and the resulting fragment ligated into *Eco*RI digested integration vector pJC1112 [28]. The resulting plasmid, pJC1112-*prom-cidA-gfp*, was transformed into *S*. *aureus* RN9011 (RN4220 containing the integrase plasmid pRN7023) [28] by electroporation. The plasmid was integrated onto the *S*. *aureus* RN9011 chromosome within *S*. *aureus* pathogenicity island 1 (SaPI1) creating the *S*. *aureus* strain designated SAPI1::*cidA*-*gfp*.

### Oligonucleotides

All oligonucleotides used in this study were generated by Integrated DNA Technologies (Skokie, IL), the nucleic acid sequence of each is available upon request.

## Results

### Identification and prioritization of putative RNA-based thermosensors in *S*. *aureus*

The annotated genome of *S*. *aureus* USA300 (accession number CP000255) was used to predict the location and sequence of putative cis-encoded RNA-based thermosensors. Specifically, the nucleic acid sequences surrounding the translation start site of each annotated open reading frame (-70nt to +30nt) were collected, and the secondary structure of the corresponding 100 nucleotide molecules was predicted by RNAfold analysis with the default settings in place. The resulting structures were analyzed and filtered to identify those in which the region likely to harbor the SD (-20nt to -6nt relative to the translational start site) was predicted to be wholly or partially sequestered within a stem-loop structure. This preliminary screen identified 277 putative RNA-based thermosensors in the *S*. *aureus* USA300 genome (S1 Table).

RNA-based thermosensors in bacteria often regulate the expression of virulence-associated genes [11, 14]. The list of 277 putative *S*. *aureus* regulators was examined to identify those located immediately upstream of genes encoding known secreted or cell wall associated virulence factors. This analysis resulted in a list of 18 putative virulence-associated ribo-regulators. Next, additional *in silico* analyses were used to further narrow down and prioritize the list of putative virulence-associated *S*. *aureus* thermosensors, prioritizing those most likely to be functional regulators. Identified sequences were compared to previously collected RNAseq datasets [32] in order to eliminate those located within transcripts that are either not detected or detected at very low levels in these datasets (5 candidates eliminated) and to adjust/modify the predicted 5’ end of the associated transcript. Secondary structures of the revised 5’ UTR sequences were predicted by mfold, and those with no significant structure (ΔG less than or equal to -0.1) were eliminated (3 candidates). Finally, the remaining 10 putative thermosensors were prioritized based on the following criteria (i) belonging to the well-characterized family of FourU RNATs [33], (ii) the likely SD is predicted to be within a hairpin structure, and (iii) a difference in the ΔG value of the predicted secondary structure at 25°C and 37°C, suggesting the presence of a temperature-responsive secondary structure (Table 2).

**Table 2:** Prioritized putative *S*. *aureus* RNA-based thermosensors.

Priority rank	Name	Gene function	ΔG at 25°C	ΔG at 37°C	ΔΔG	4U RNAT
1	*sspB*	Staphopain B	-12.7	-7.9	4.7	+
2	*ssaA*	SsaA-like protein	-10.5	-6.9	3.6	+
3	*cidA*	Holin-like protein	-22.4	-14.6	7.8	-
4	SAUSA300_0651	SsaA-like protein	-17.0	-10.9	6.1	-
5	SAUSA300_1056	Fibrinogen-binding protein precursor	-17.5	-11.7	5.8	-
6	*clfB*	Clumping factor B	-13.2	-8.8	4.42	-
7	SAUSA300_1052	Complement inhibitory protein	-4.7	-2.8	1.9	-
8	*aur*	Aureolysin	-10.9	-7.0	3.87	-
9	SAUSA300_1973	Truncated beta-hemolysin	-18.4	-10.8	7.6	-
10	SAUSA300_0223	Complement inhibitor protein	-4.5	-2	2.5	-

### Two predicted *S*. *aureus* FourU RNATs do not confer temperature dependent regulation

The top two candidate thermosensors, both putative 4U RNATs, are located within the 5’ UTRs of *sspB* and *ssaA* (Table 2). The ability of putative *sspB* and *ssaA* 4U RNATs to confer temperature dependent regulation was investigated using a reporter plasmid-based approach [16, 17]. By definition, nucleic acid sequences composing a functional RNAT would be sufficient to confer temperature-dependent post-transcriptional regulation onto the expression of a reporter gene. Furthermore, a functional RNAT would be expected to mediate regulation within an ectopic bacterial host, as regulation is independent of additional cellular factors. These predictions were tested directly by the construction and characterization of translational reporter plasmids. Nucleic acid sequences composing the putative *sspB* and *ssaA* RNATs were cloned between the constitutive plasmid promoter and the RBS-less *gfp* reporter gene on plasmid pXG10 [29], generating p*sspB*-UTR and p*ssaA*-UTR respectively. Such cloning places expression of the *gfp* reporter gene under control of the constitutive plasmid promoter and subject to any post-transcriptional regulation mediated by the cloned *S*. *aureus* sequences, in this case a putative *S*. *aureus* RNAT.

**Fig 1:**
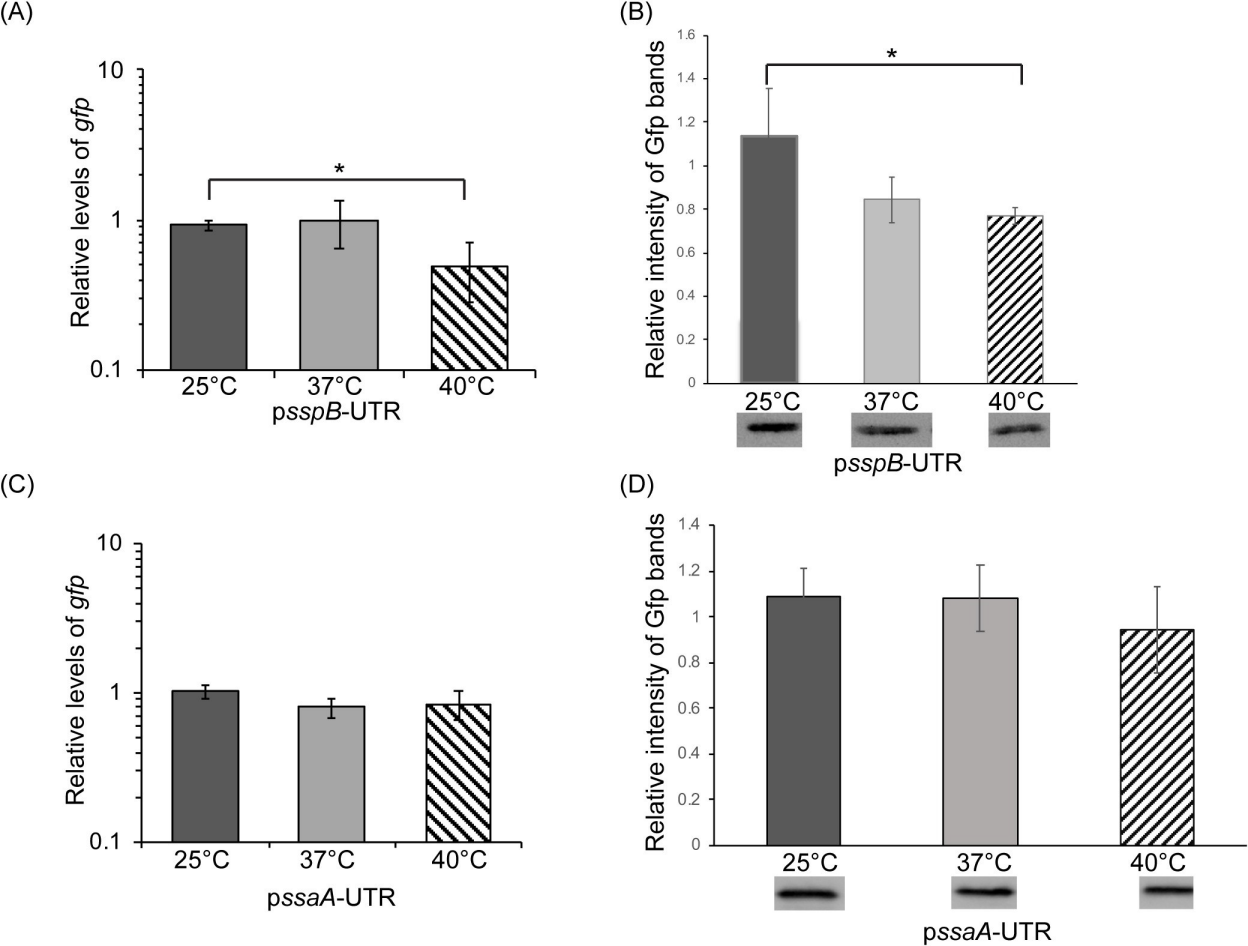
The *sspB* and *ssaA* 5’ UTRs are not sufficient to confer temperature-dependent post-transcriptional regulation. (A&C) Quantitative real-time PCR analysis of the relative amounts of *gfp* transcript present in *E*. *coli* carrying the indicated reporter plasmid following growth to the mid-logarithmic phase at 25°C, 37°C or 40°C. Using the ΔΔCt method, *gfp* levels are normalized to that of *rrsA* in each sample and are expressed relative to *gfp* levels within a single 25°C sample. (B&D) Western blot analysis of the relative amounts of Gfp present in *E*. *coli* carrying the indicated reporter plasmid following growth to the mid-logarithmic phase at 25°C, 37°C or 40°C. Data are expressed relative to a single 25°C sample on the same blot. Images of the blots used to generate these data are shown in S1 Fig. All data shown are the average of analyses completed in biological triplicate and error bars represent one standard deviation. * indicates a statistically relevant difference where p<0.05.

Once constructed and sequence verified, p*sspB*-UTR and p*ssaA*-UTR were independently introduced into *E*. *coli* DH5α. The relative amount of *gfp* and Gfp was measured following growth of each resulting reporter strain at various temperatures by quantitative real-time PCR (qRT-PCR) and western blot analyses, respectively. A difference in the expression pattern observed for the *gfp* transcript and the Gfp protein would indicate that the reporter gene is subject to temperature-dependent post-transcriptional regulation. Surprisingly, neither the putative RNAT within *sspB*, nor that within *ssaA* was able to confer temperature-dependent post-transcriptional regulation onto the expression of the *gfp* reporter gene (Fig 1 and S1 Fig). As the selected putative RNATs each contain features conserved amongst members of the well-characterized FourU family of RNATs [34], these data highlight the intrinsic limitations of *in silico* predictions and the importance of continued experimental investigation into this class of regulators.

### The *cidA* 5’ UTR is sufficient to confer temperature-dependent post-transcriptional regulation

**Fig 2:**
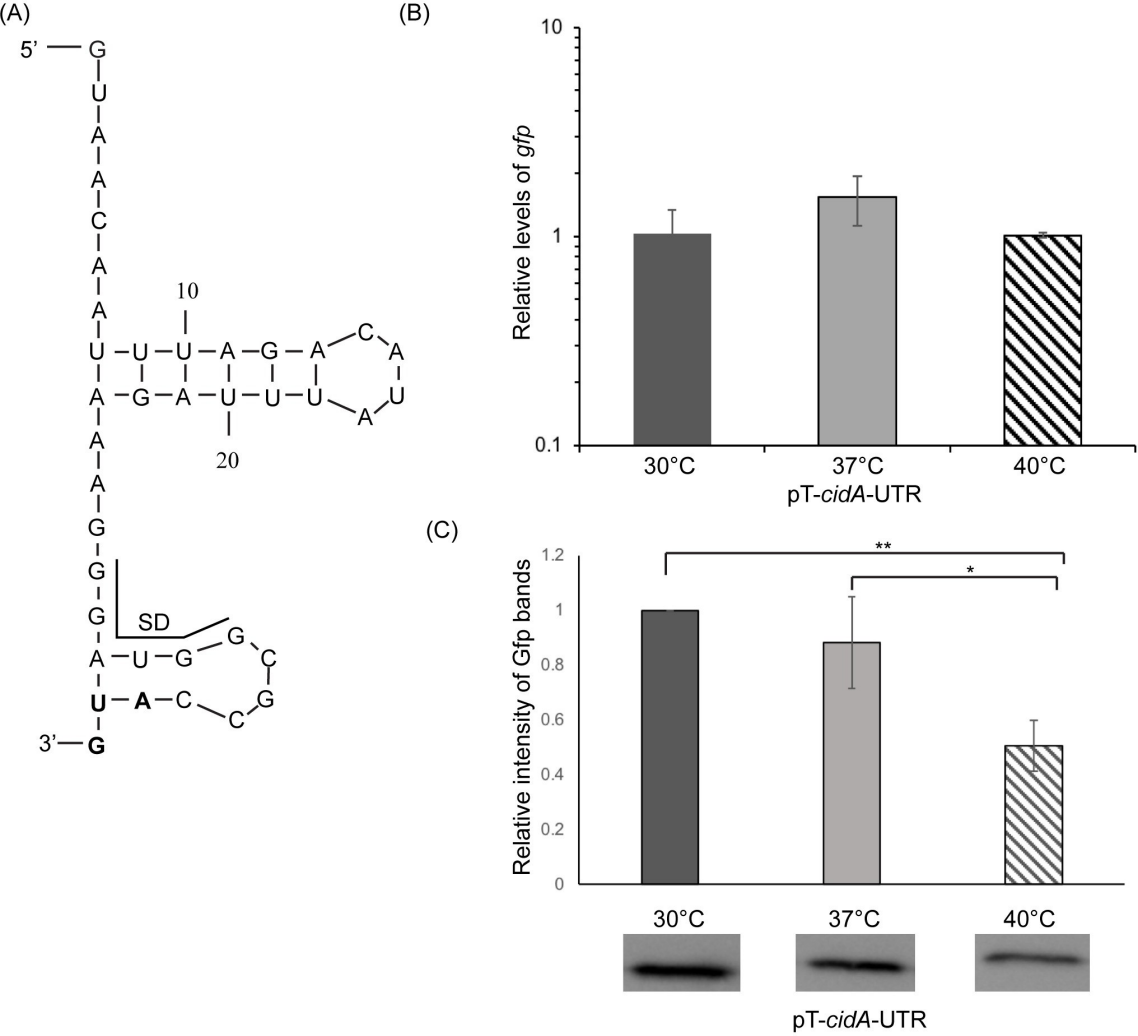
The *cidA* 5’ UTR is sufficient to confer temperature-dependent post-transcriptional regulation. (A) The nucleotide sequence and putative structure within the 36nt long *cidA* 5’ UTR as predicted by M-fold analysis [36]. The likely SD region is indicated with the black line and the translational start site in bold. (B) Quantitative real-time PCR analysis of the relative amounts of *gfp* transcript present in the *E*. *coli* reporter strain following growth to the mid-logarithmic phase at 30°C, 37°C, and 40°C. Using the ΔΔCt method, *gfp* levels are normalized to that of *rrsA* in each sample and expressed relative to *gfp* levels within a single 30°C sample. (C) Western blot analysis of the relative amounts of Gfp present in the same strains used for *gfp* measurement above. Data are expressed relative to the 30°C sample in each set. Images of the blots used to generate these data are shown in S2 Fig. All data shown are the average of analyses completed in biological triplicate and error bars represent one standard deviation. * indicates a statistically relevant difference where p<0.05, and ** indicates p<0.01.

The functionality of the putative RNA-based thermosensor within *cidA* was the next to be experimentally investigated. Unlike the first two putative regulators tested, the potential regulatory region within the *cidA* 5’ UTR is not predicted to be a 4U RNAT (Table 2). The 5’ UTR of *S*. *aureus cidA* has been experimentally determined to be 36nt long [35], and is predicted by mfold analysis [36] to form two hairpin structures, with the second partially sequestering the likely SD (Fig 2A).

To experimentally test the ability of the putative *cidA* thermosensor to confer temperature-dependent post-transcriptional regulation, the 36nt sequence composing the *cidA* 5’ UTR along with the translational start codon was cloned between the constitutive pLtetO-1 promoter and the *gfp* reporter gene of plasmid pXG-10 [29] as detailed above. Designated pT-*cidA*-UTR, the translational reporter plasmid was sequence verified and introduced into *E*. *coli*. The resulting reporter strain was cultured to the mid-logarithmic phase of growth at varied temperatures, and the relative abundance of *gfp* transcript and Gfp protein measured by qRT-PCR and western blot analyses, respectively. For this and the remainder of the assays in this study, analysis at 25°C is replaced by that at 30°C, since this temperature more closely mimics that encountered by *S*. *aureus* during carriage in the external nares or on the skin.

While there is no significant change in the relative amount of *gfp* transcript measured at any of the tested temperatures (Fig 2B), a significant reduction in the amount of Gfp protein is measured following growth of the strain at 40°C as compared to that measured at 30°C or 37°C (Fig 2C and S2 Fig). Differing amounts of protein generated from equivalent levels of transcript indicate that the *gfp* reporter gene of pT-*cidA*-UTR is subject to temperature-dependent post-transcriptional regulation, regulation mediated by the cloned sequence. Given that the cloned sequences mediate increased target gene expression at lower temperatures rather than at higher temperatures, it is clear that the identified regulatory element within the *cidA* 5’ UTR does not function as a typical bacterial RNAT, a finding that highlights the potential diversity among RNA-based thermosensors.

### Sequences upstream of the *cidA* open reading frame are sufficient to confer temperature dependent post-transcriptional regulation in *S*. *aureus*

Given the unexpected finding that the *cidA* thermosensor facilitates increased target gene expression at lower temperatures rather than higher temperatures as anticipated, it was essential to characterize the regulatory activity of this element within the context of the *S*. *aureus* genome. In the absence of anti-CidA antibodies required to directly measure the impact of temperature on CidA production, a *gfp*-reporter construct was generated and integrated into the *S*. *aureus* genome. The translation start site of *cidA* along with the 282 preceding bases (including the promoter and 5’ UTR) was cloned upstream of an ATG-less *gfp* reporter gene, and the construct integrated at the SAPI1 site of the *S*. *aureus* genome, creating the reporter strain SAPI1::*cidA*. Expression of the integrated reporter *gfp* gene from SAPI1::*cidA* is subject to all regulation mediated by sequences within the native *cidA* promoter and 5’ UTR regions.

**Fig 3:**
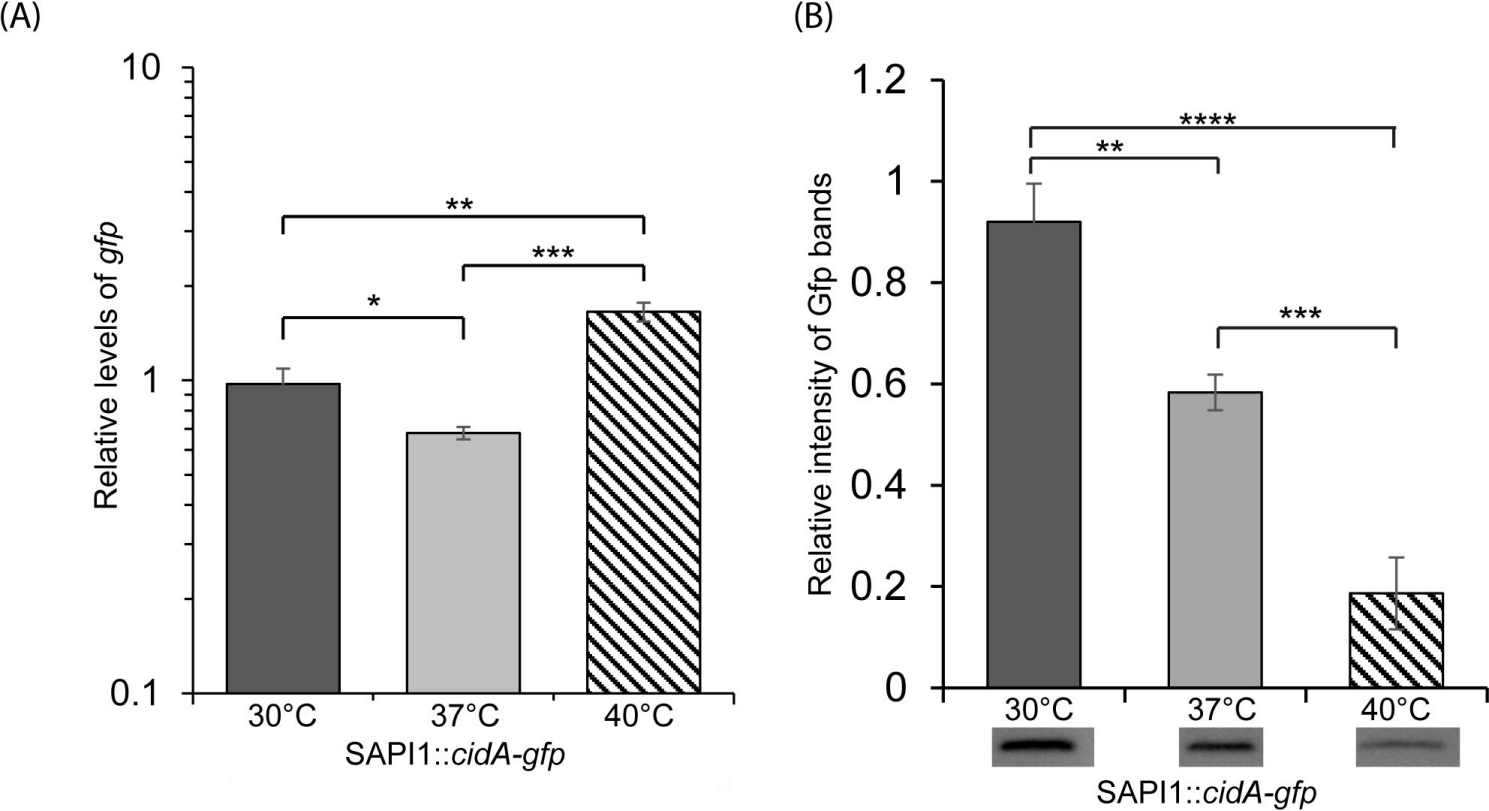
The *RNA-based* thermosensor within *cidA* is active when integrated into the *S*. *aureus* chromosome. (A) Quantitative Real-time PCR analysis of *gfp* levels present in the *S*. *aureus* reporter strain (SAPI1::*cidA*-*gfp*) following growth to the mid-logarithmic phase at 30°C, 37°C or 40°C. Using the ΔΔCt method, *gfp* levels were normalized to that of *gyrB* present in each sample and expressed relative to *gfp* levels in a single 30°C sample. (B) Western blot analyses of Gfp levels present in the same samples utilized for transcript analyses. Protein levels are expressed relative to that in a single 30°C sample. Images of the blot used to generate these data are shown in S3 Fig. All data shown are the average of analyses completed in biological triplicate and error bars represent one standard deviation. Statistically significant differences are denoted by asterisks with * indicating p<0.05, ** indicating p<0.01, *** indicating p<0.001, and **** indicating p<0.0001.

Following growth of the SAPI1::*cidA*-*gfp* reporter strain at 30°C, 37°C, and 40°C, the relative abundance of *gfp* and Gfp was measured by qRT-PCR and western blot analyses, respectively. While the relative abundance of *gfp* transcript present in the reporter strain increases 1.6-fold between 30°C and 40°C (Fig 3A), a significant decrease in the relative abundance of Gfp was observed with each incremental increase in temperature, resulting in an overall 5-fold reduction from 30°C to 40°C (Fig 3B and S3 Fig).

### Transcripts with an extended 5’ UTR constitute a minor sub-population of total *cidA* mRNA generated by *S*. *aureus*

**Fig 4:**
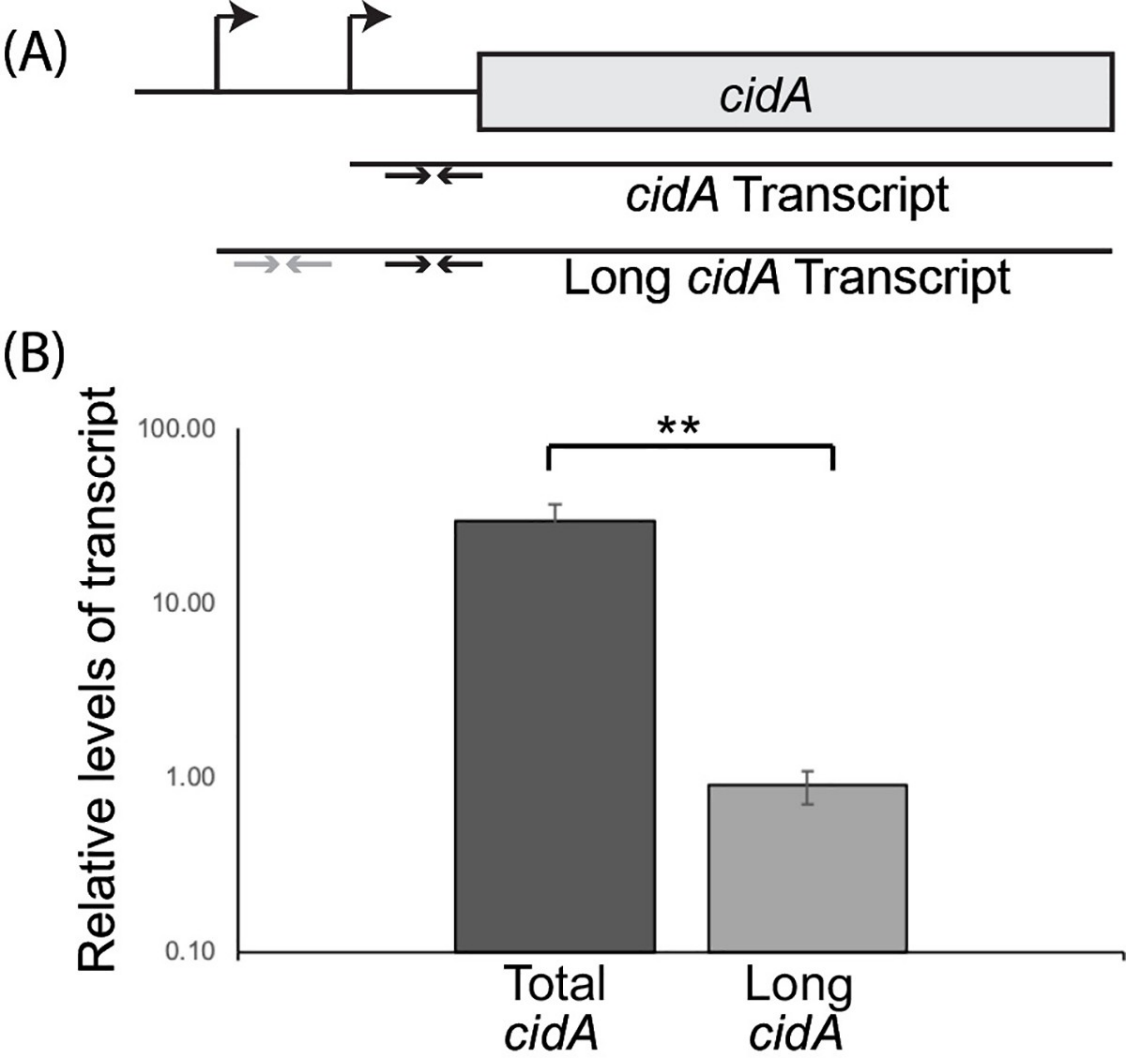
The long *cidA* transcript represents 3% of the total *cidA* transcript pool in *S*. *aureus*. (A) Schematic of the *S*. *aureus cidA* open reading frame (not drawn to scale). Each promoter is indicated by a bent arrow, and the corresponding transcripts (*cidA* and long *cidA*) are represented by black lines. The sets of black and grey arrows represent the Real-time primer sets used to detect the relative abundance of total *cidA* transcript or long *cidA* transcript, respectively. (B) Quantitative Real-time analysis of the relative abundance of total *cidA* transcript and long *cidA* transcript present in WT *S*. *aureus* cultured to the mid-logarithmic phase at 30°C. All data shown are the average of analyses completed in biological triplicate and error bars represent one standard deviation. Statistically significant differences are denoted by asterisks with ** indicating p<0.01.

Analysis of published RNA-seq data sets suggests the presence of a minor sub-population of *S*. *aureus cidA* transcripts with an extended 82nt long 5’ UTR [32]. The presence and relative quantity of this long *cidA* transcript was experimentally investigated using qRT-PCR analyses (Fig 4). For this analysis, wild-type *S*. *aureus* was cultured to the mid-logarithmic phase of growth at 30°C and the relative abundance of total *cidA* transcript and long *cidA* transcript measured using primers designed to detect each (Fig 4A). The long *cidA* transcript was detected and determined to represent 3of the total pool of *cidA* transcript in *S*. *aureus* under the conditions tested (Fig 4B). These data are consistent with the previously published RNAseq study[32], confirming the existence of a minor population of *S*. *aureus cidA* transcript containing an extended 5’ UTR.

### Additional sequences with an extended *cidA* 5’ UTR amplify the regulatory activity of the thermosensor

**Fig 5:**
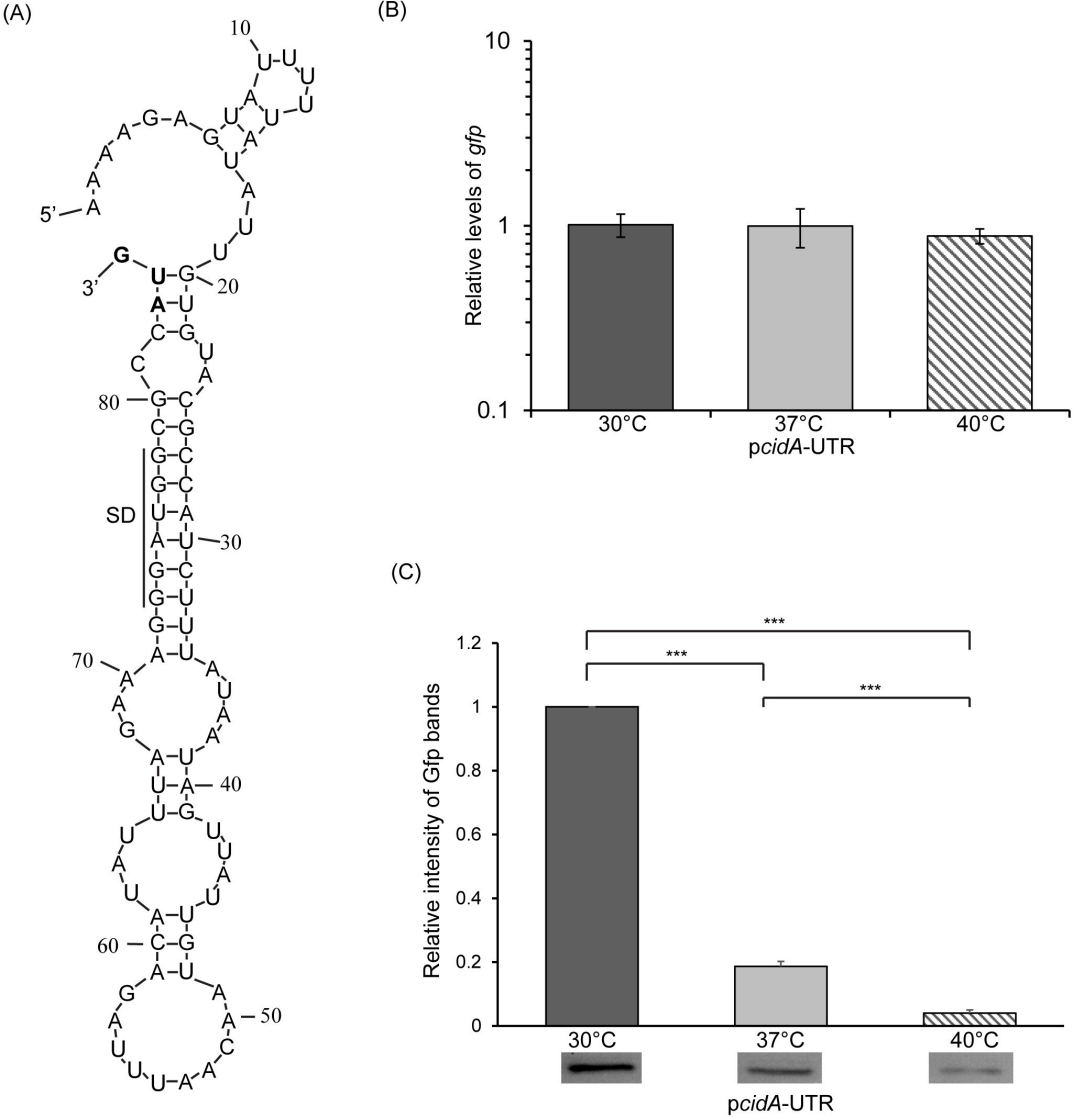
The extended *cidA* 5’ UTR is sufficient to confer temperature-dependent post-transcriptional regulation. (A) Nucleic acid sequence and putative structure within the extended 82 nt long *cidA* 5’ UTR as predicted by M-fold analysis [36]. The likely SD is indicated with a black line and the translational start codon is bolded. (B) Quantitative real-time PCR analysis of relative amounts of *gfp* transcript present in the reporter strain following growth to the mid-logarithmic phase at 30°C, 37°C, and 40°C. Using the ΔΔCt method, *gfp* levels are normalized to that of *rrsA* present in each sample and expressed relative to *gfp* levels that in a single 30°C sample. (C) Western blot analysis of the relative amounts of Gfp present in the same samples used for *gfp* measurement. Data are expressed relative to the 30°C sample in each set. Images of the blots used to generate these data are shown in S4 Fig. All data shown are the average of analyses completed in biological triplicate and error bars represent one standard deviation. Statistically significant differences are denoted by asterisks with *** indicating p<0.001.

M-fold analysis [36] of the extended 82 nt *cidA* 5’ UTR predicts the formation of a structure in which the likely SD is occluded within a stem-loop structure (Fig 5A). As compared to that within the 36nt 5’ UTR, the likely SD is more extensively base-paired in the predicted structure of the 82nt 5’ UTR, a feature that would be expected to result in further reduced expression of the regulated gene at non-permissive temperatures. To experimentally investigate the potential of sequences within the longer 5’ UTR to confer temperature-dependent post-transcriptional regulation, a second *cidA* translational reporter was constructed. Specifically, sequences composing the 82nt long *cidA* 5’ UTR along with the translation start site were cloned between the constitutive pLtetO-1 promoter and ATG-less *gfp* reporter gene of pXG-10 [29] to generate the translational reporter plasmid p*cidA*-UTR. *E*. *coli* carrying p*cidA*-UTR was cultured to the mid-logarithmic growth phase at 30°C, 37°C and 40°C prior to the parallel measurement of the relative amount of *gfp* transcript and Gfp protein present as detailed above. While an equivalent amount of *gfp* transcript is present following growth of the reporter strain at each temperature tested (Fig 5B), each incremental increase in growth temperature results in a significant decrease in the amount of Gfp protein measured; resulting in a 25-fold reduction between 30°C and 40°C (Fig 5C and S4 Fig). Together these data clearly indicate that sequences within the 82nt long *cidA* 5’ UTR are sufficient to confer post-transcriptional temperature-dependent regulation and are consistent with the conclusion that this region houses a functional RNA-based thermosensor.

As compared to that mediated by sequences within the short *cidA* 5’ UTR (Fig 2), sequences of the longer *cidA* UTR confer greater regulatory activity, and this regulation more closely resembles that observed from the integrated native reporter (Fig 3). For these reasons, the longer molecule was selected for further characterization.

### Site-specific mutagenesis alters the activity of the *cidA* thermosensor

**Fig 6:**
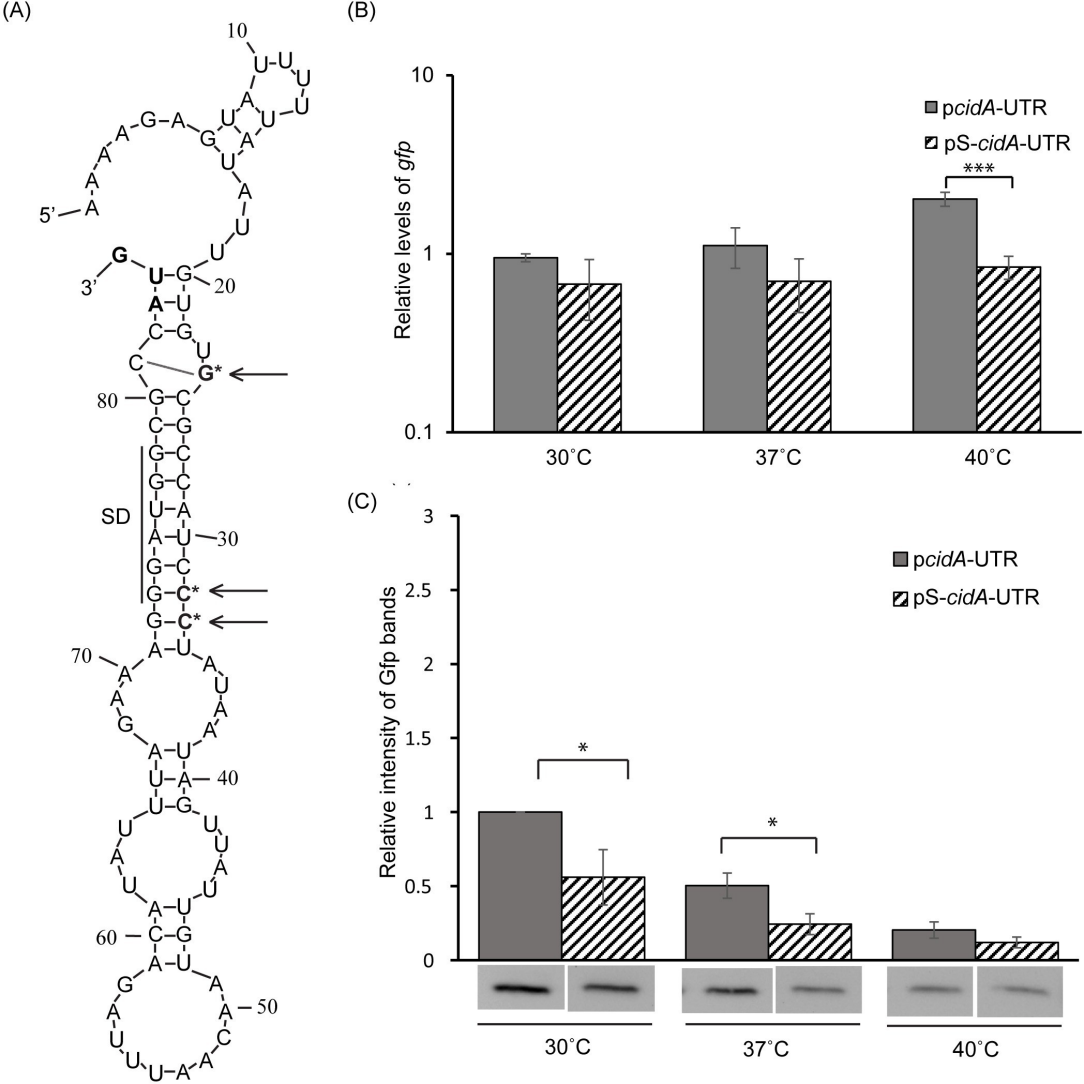
Site-specific mutations predicted to stabilize the putative inhibitory structure within the *cidA* 5’ thermosensor reduce target gene expression at permissive temperatures. (A) Nucleic acid sequence and predicted structure of the 82nt long *cidA* 5’ UTR containing site-specific mutations at positions 24, 32 and 33. These mutations, indicated by an arrow and asterisk, are predicted to stabilize the SD sequestering structure within the *cidA* thermosensor. (B) Quantitative real-time PCR analysis of the relative abundance of *gfp* transcript present in *E*. *coli* DH5α cells carrying the WT (p*cidA*-UTR) or mutant (pS-*cidA*-UTR) translational reporter plasmids following growth of the strains to the mid-logarithmic phase at 30°C, 37°C and 40°C. Using the ΔΔCt method, *gfp* levels are normalized to that of *rrsA* present in each sample and expressed relative to *gfp* levels in a single p*cidA*-UTR sample at 30°C. (C) Western blot analyses of the relative abundance of Gfp protein in the same samples as used above. Gfp levels are expressed relative to the 30°C sample in each set. Images of the blots used to generate these data are shown in S5 Fig. All data shown are the average of analyses completed in biological triplicate and error bars represent one standard deviation. Statistically significant differences are denoted by asterisks with * indicating p<0.05 and *** indicating p<0.001.

Site-specific mutagenesis was used to further investigate the existence of a functional RNA-based thermosensor within the 82nt long *cidA* 5’ UTR. The site-specific mutations incorporated are predicted to either stabilize or destabilize the inhibitory, SD sequestering structure within the regulatory element, while not altering the overall structure. The first site-specific mutations incorporated were those predicted to stabilize the potential SD sequestering hairpin within the *cidA* thermosensor. In this mutant element, the adenine at position 24 (relative to the 5’ terminus of the transcript) was changed to a guanosine, and the uracils at positions 32 and 33 (relative to the 5’ terminus of the transcript) were changed to cytosines (Fig 6A). Such stabilizing mutations would be expected to result in decreased reporter protein production at permissive temperatures. As expression from the WT reporter is very low at the non-permissive temperature of 40°C (Fig 5C), a further decrease may not be seen as a result of stabilizing mutations within the thermosensor. The mutated 5’ UTR was cloned into pXG-10 [29] as detailed above, generating the translational reporter plasmid pS-*cidA*-UTR.

To test the effect of the site-specific mutations on the regulatory activity of the *cidA* thermosensor, *E*. *coli* carrying the wild-type (p*cidA*-UTR) or mutated reporter plasmid (pS-*cidA*-UTR) were cultured to the mid-logarithmic phase at 30°C, 37°C or 40°C and the relative amounts of *gfp* transcript and Gfp protein present in each sample was measured as detailed above. While the introduction of site-specific stabilizing mutations had no impact on the relative abundance of *gfp* produced at 30°C and 37°C (Fig 6B), significantly less Gfp protein was present in the strains carrying the mutant reporter plasmid at each of these temperatures (Fig 6C and S5 Fig). Despite significantly more *gfp* transcript measured in the strain carrying the wild-type reporter plasmid (p*cidA*-UTR), an equivalently low level of Gfp protein was measured in each reporter strain following growth at 40°C, indicating decreased translational efficiency from the mutant transcript. Given the exceptionally low level of Gfp produced in the strain carrying the wild-type reporter at the non-permissive temperature of 40°C (Figs 5C and 6C), it is not surprising that stabilizing site-specific mutations did not result in a significant reduction of Gfp production.

**Fig 7:**
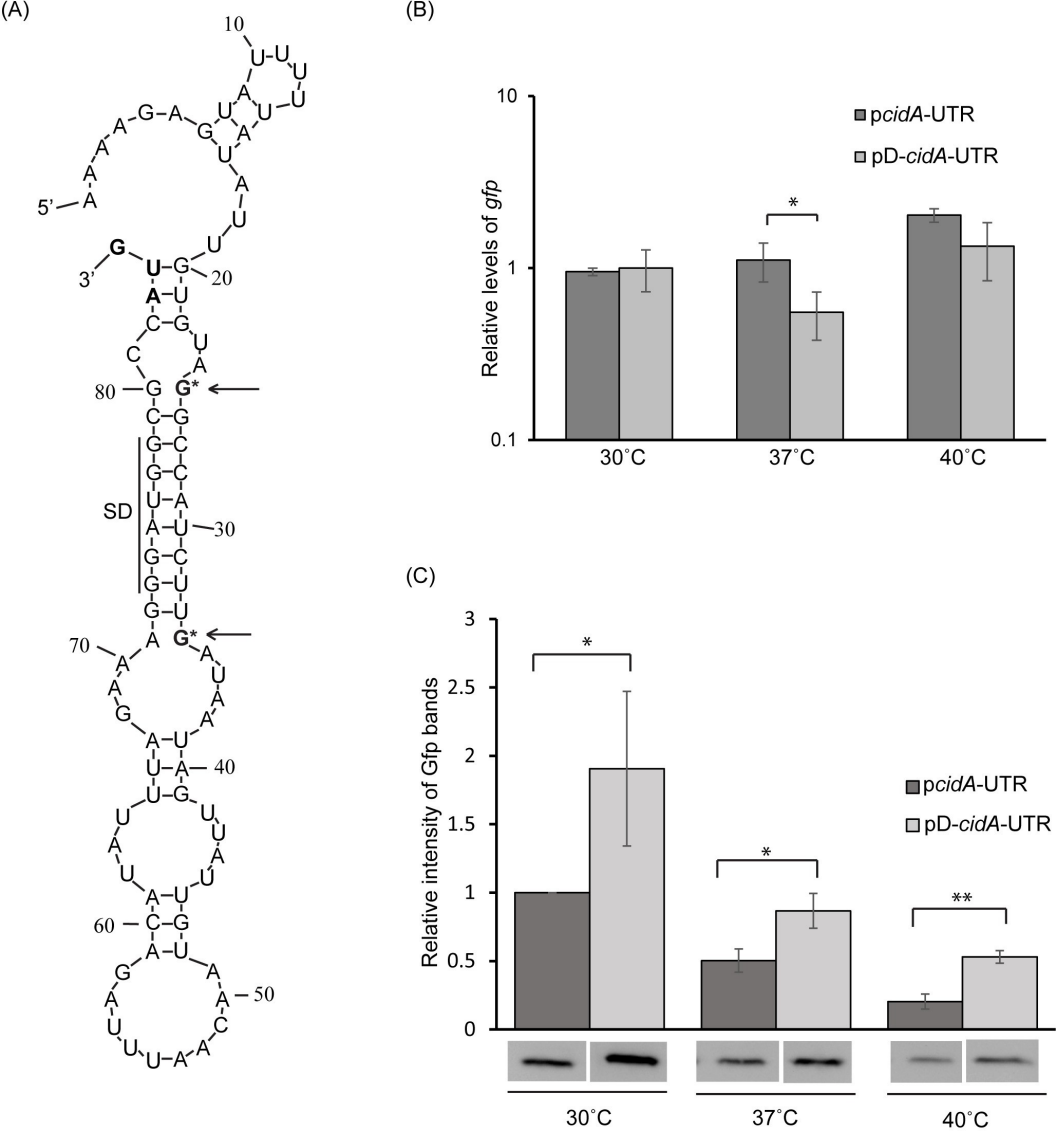
Site-specific mutations predicted to destabilize the inhibitory structure within the *cidA* thermosensor increase target gene expression. (A) Nucleic acid sequence and predicted structure of the extended *cidA* 5’ UTR containing mutations at positions 25 and 34. These mutations, indicated by an arrow and asterisk, are predicted to destabilize the inhibitory, SD sequestering structure within the *cidA* RNA thermosensor. (B) Quantitative Real-time PCR measuring the relative abundance of *gfp* in *E*. *coli* carrying the WT (p*cidA*-UTR) or mutant (pD-*cidA*-UTR) reporter plasmid following growth at 30°C, 37°C and 40°C. Using the ΔΔCt method, *gfp* levels are normalized to that of *rrsA* present in each sample and expressed relative to *gfp* levels in a single p*cidA*-UTR sample at 30°C. (C) Western blot analyses of the relative abundance of Gfp in the same samples used above. Gfp levels are expressed relative to the 30°C sample in each set. Images of the blots used to generate these data are shown in S5 Fig. All data shown are the average of analyses completed in biological triplicate and error bars represent one standard deviation. Statistically significant differences are denoted by asterisks with * indicating p<0.05, and ** indicating p<0.01.

The second set of site-specific mutations incorporated into the extended *cidA* 5’ UTR were those predicted to destabilize the SD sequestering structure within the *cidA* thermosensor. In this mutated element the cytosine and uracil at positions 25 and 34 respectively (relative to the 5’ terminus of the molecule) were mutated to guanosines (Fig 7A). Such destabilizing mutations are expected to result in increased production of the regulated protein at non-permissive temperatures. As expression is already de-repressed at the permissive temperature of 30°C (Fig 5C), destabilization of the structure within the thermosensor is unlikely to result in increased target gene expression at this temperature. The mutant *cidA* 5’ UTR was cloned into pXG-10 [29] as detailed above, generating the translational reporter plasmid pD-*cidA*-UTR.

To test the impact of the destabilizing mutations on the regulatory activity of the *cidA* thermosensor, the relative abundance of *gfp* and Gfp present in *E*. *coli* carrying the mutant reporter (pD-*cidA*-UTR) was measured at 30°C, 37°C or 40°C, and compared to that generated from the WT reporter (p*cidA*-UTR) at each temperature. The relative abundance of *gfp* transcript and Gfp protein present in each reporter strain was measured as detailed above. While *gfp* levels were equivalent (30°C and 40°C) or slightly lower (37°C) (Fig 7B), Gfp levels were significantly higher in the strain carrying the mutated reporter (pD-*cidA*-UTR) as compared to that in the strain carrying the WT reporter (p*cidA*-UTR) at all temperatures tested (Fig 7C and S5 Fig.). Together, these data demonstrate that a functional thermosensor is housed within the 5’ UTR of *S*. *aureus cidA*; the first such regulator identified in this species.

## Discussion

Data presented within this study are the first to identify and experimentally characterize an RNA-based thermosensor in the genus *Staphylococcus*, a genus known to experience varied temperatures during states of carriage verses invasive infection. Essential to this designation are the findings that sequences within the *cidA* 5’ UTR are sufficient to confer temperature-dependent post-transcriptional regulation (Figs 2 and 5), and that site-directed mutagenesis results in predictable alterations in regulatory activity (Figs 6 and 7).

Despite being identified using *in silico* approaches designed to identify traditional RNATs expected to mediate increase target gene expression at relatively high temperature, the experimental characterizations demonstrate that the *cidA* thermosensor mediates increased target gene expression at relatively low temperatures. While unconventional, the observed expression pattern of the integrated chromosomal reporter (Fig 3) clearly demonstrates that *cidA* thermosensor-mediated temperature-dependent post-transcriptional regulation is a biologically relevant phenomenon. In addition to providing insight into *S*. *aureus* gene expression, this unexpected finding brings to light two important considerations to the field moving forward. Frist, the disconnect between the *in silico* predictions of structure, and thus function of the *cidA* thermosensor with that revealed by molecular characterization highlights the inherent limitations of *in silico* predictions in the field of RNA structure/function. Structural predictions are not perfect. Given the observed activity of the *cidA* thermosensor, it is unlikely that the predicted structures are present in the native RNA molecules. The precise structure within the *cidA* thermosensor and how this structure mediates that observed regulatory activity is the focus of ongoing investigation. Second, these studies highlight the diversity of function among bacterial RNA-based thermosensors. Not all thermosensors function like traditional RNATs. Characterizing the diversity of function within bacterial RNA-based thermosensors is an exciting frontier in the field.

Given the observed regulatory activity, it is unlikely that the *cidA* thermosensor regulates target gene expression via the mechanism conserved amongst RNATs characterized to date. The established understanding of the molecular mechanism underlying RNAT-mediated regulation is that there is a gradual, “zipper-like” destabilization of a temperature-sensitive inhibitory structure that when formed, occludes the ribosomal binding site [12]. Such regulation results in decreased target gene expression at relatively low temperatures (25°-30°C) when the SD-sequestering structure is relatively stable, and increased target gene expression at relatively high temperatures (37°C-40°C) when the inhibitory structure is relatively unstable. The molecular mechanism underlying the regulatory activity of the *S*. *aureus cidA* thermosensor is yet to be characterized. Similar to the thermosensor controlling the expression of the *E*. *coli* cold-shock gene *cspA* [37], it is possible that RNA within the *cidA* 5’ UTR assumes mutually exclusive structures with different ribosomal binding efficiencies at different temperatures. The observed impact of site-specific mutations within the *cidA* thermosensor is consistent with this potential mechanism of regulation. While designed to stabilize (Fig 6) or destabilize (Fig 7) the putative SD sequestering structure within the *cidA* thermosensor, the introduced mutations would likely impact the ability of the element to assume an alternative structure. Specifically, if a “closed” structure similar to that predicted by mfold analysis (Fig 5) is favored at relatively warm temperatures, site-specific mutations that stabilize the central structural component would likely prevent the formation of an alternative “open” configuration, resulting in the observed decrease in target gene expression (Fig 6). Alternatively, site-specific mutations that weaken the central structure within the *cidA* thermosensor would likely increase the rate at which an alternative structure is assumed, thus promoting target gene expression at non-permissive temperatures (Fig 7). Further studies will be aimed at elucidating the molecular mechanism of the underlying regulatory activity of the *cidA* thermosensor.

In addition to identifying a functional thermosensor within the *cidA* transcript, these studies have revealed the existence of a longer *cidA* transcript containing an extended 5’ UTR (Fig 4). While sequences composing both forms of the *cidA* 5’ UTR are sufficient to confer temperature-dependent regulation, clear differences are seen in the pattern and extent of regulation that is mediated by each (Figs 2 and 5). The significance of the relative abundance and differential thermo-regulation mediated by the long and short *cidA* transcripts will be the subject of future investigations.

The data presented within suggest that in *S*. *aureus* there is increased CidA production at lower temperatures. While the precise mechanism of action of CidA in *S*. *aureus* is unknown, it is thought to promote a pro-survival phenotype in *S*. *aureus* stationary phase cells, most likely by increasing turnover of the cell death-inducing protein, CidC [25]. CidC-mediated cell death is thought to play an important role in *S*. *aureus* biofilm formation. In the post-exponential phase of growth, localized acetate buildup leads to cytoplasmic acidification and subsequent lysis of a subpopulation of cells in a CidC-dependent manner. Cell lysis, and the subsequent release of extracellular DNA, aids in biofilm attachment and formation, consequently CidC has a positive influence on biofilm formation. CidA in counteracting the effect of CidC has a pro-survival phenotype. The biological significance of increased CidA production at lower temperatures is unclear: however, it is possible that under conditions of decreased temperature (i.e. the skin and nares) the bacteria experience increased cytoplasmic acidification, either due to acetate buildup or some other means. In this case, increased CidA production would counteract the pro-cell death activity of CidC and facilitate increased bacterial survival. Alternatively, it is also possible that CidA has some as of yet unidentified function in the cell that is required in low temperature environments.

Bacterial RNA-based thermosensors have primarily been characterized in Gram-negative bacteria, with little known about the distribution and significance of this important class of ribo-regulators in Gram-positive species. In addition to being the first identified in *Staphylococcus*, the *cidA* RNA-based thermosensor is just the second such regulator to be identified in any Gram-positive species [15]. Of the three putative RNA-based thermosensors experimentally investigated in this study (*sspB*, *ssaA* and *cidA*), just one was confirmed to be a functional regulator. It is noteworthy that while similar characteristics were sought, the success rate of predicting RNA-based thermosensors in *S*. *aureus* is less than in that of Gram-negative species such as *Shigella dysenteriae* and *E*. *coli* [16, 17]. Such a discrepancy opens the door to the existence of previously unappreciated variation among bacterial thermosensors, variation that will be fully appreciated only by continued investigations in both Gram-negative and Gram-positive bacteria.

## Supporting information

S1 Fig Gel images used to generate western blot data presented in Fig 1. (A) Whole gel image of triplicate Gfp western blot using *E*. *coli* (p*sspB*-UTR) cultured at the indicated temperatures. The box indicates the representative bands that are shown as inserts in Fig 1B. (B) Whole gel image of triplicate Gfp western blot using *E*. *coli* (p*ssaA*-UTR) cultured at the indicated temperatures. The box indicates the representative bands that are shown as inserts in Fig 1D. Bands seen under the main band in each lane appear to have the same relative density as Gfp and are consistent with the presence of Gfp degradation products in these samples.
(TIF)

S2 Fig Gel images used to generate western blot data presented in Fig 2. Two gels containing Gfp western blots performed in triplicate with *E*. *coli* (pT*cidA*-UTR) cultured at the indicated temperatures. Data resulting from growth of the reporter strain at 30°C, 37°C and 40°C were selected for inclusion in the presented study. The box indicates the representative bands that are shown as inserts in Fig 2C.
(TIF)

S3 Fig Gel images used to generate western blot data presented in Fig 3. Whole gel image of triplicate Gfp western blot using *S*. *aureus* SAPI1::*cidA-gfp* cultured at the indicated temperatures. The box indicates the representative bands that are shown as inserts in Fig 6B. This blot was cut prior to exposure to the primary antibody in order to eliminate antibody absorption by Protein A.
(TIF)

S4 Fig Gel images used to generate western blot data presented in Fig 5. Two gels containing Gfp western blots performed in triplicate with *E*. *coli* (p*cidA*-UTR) cultured at the indicated temperatures. Data resulting from growth of the reporter strain at 30°C, 37°C and 40°C were selected for inclusion in the presented study. The box indicates the representative bands that are shown as inserts in Fig 3C.
(TIF)

S5 Fig Gel images used to generate western blot data presented in Figs 6 and 7. Three gels containing Gfp western blots performed in triplicate with *E*. *coli* (p*cidA*-UTR), *E*. *coli* (pS-*cidA*-UTR) and *E*. *coli* (pD-*cidA*-UTR) cultured at the indicated temperatures. The box indicates the representative bands that are shown as inserts in Figs 4C and 5C.
(TIF)

S1 Table 277 putative thermosensors identified by *in silico* analysis of the *S*. *aureus* genome. (TIF)
