# Supplementary figures and images for: An unconventional RNA-based thermosensor within the 5’ UTR of Staphylococcus aureus cidA
Source: PLoS One. 2019 Apr 1;14(4):e0214521. doi: 10.1371/journal.pone.0214521 (PMC6443170; doi:10.1371/journal.pone.0214521)

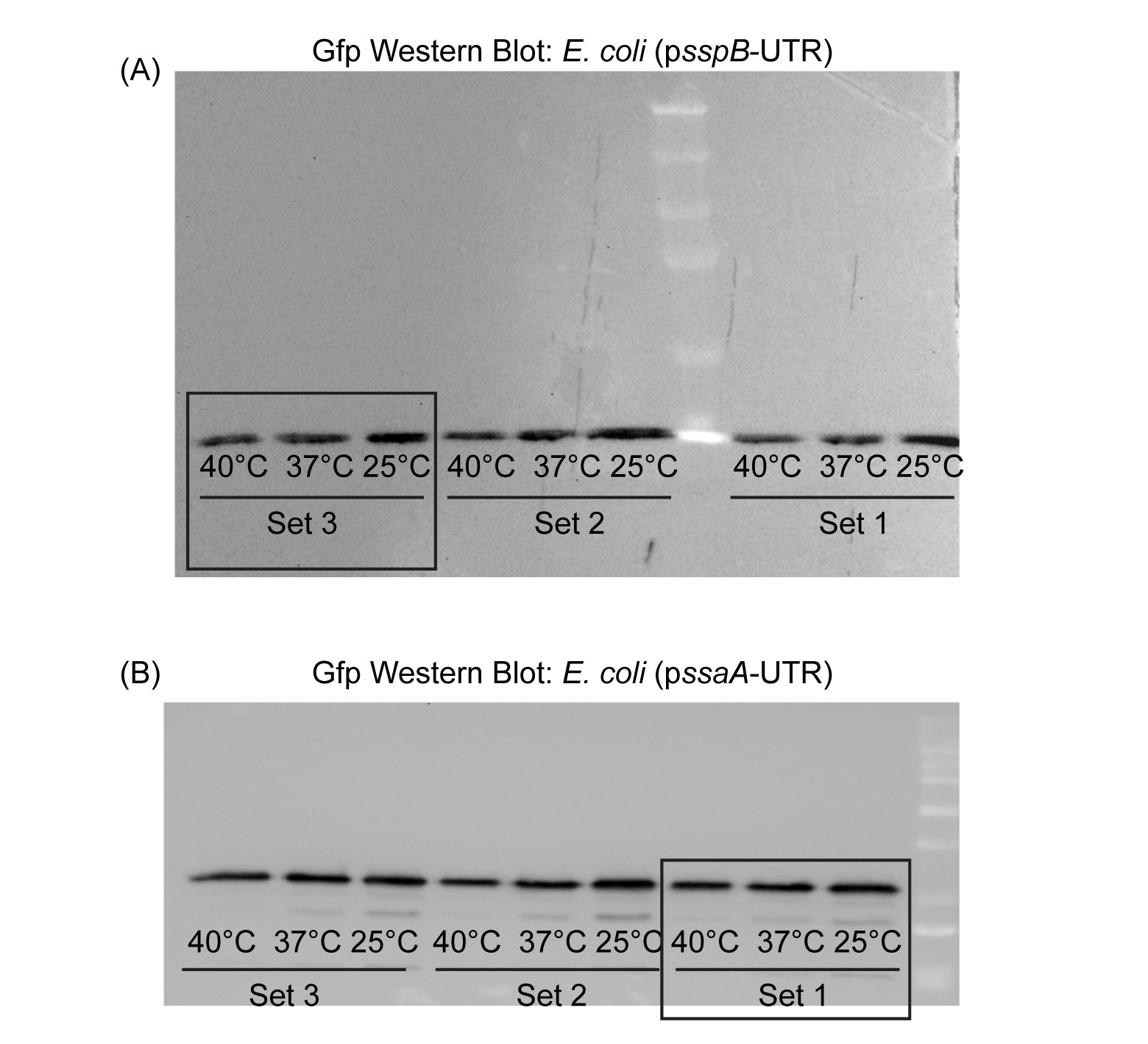

Supplement: S1 Fig — (A) Whole gel image of triplicate Gfp western blot using E. coli (psspB-UTR) cultured at the indicated temperatures. The box indicates the representative bands that are shown as inserts in Fig 1B. (B) Whole gel image of triplicate Gfp western blot using E. coli (pssaA-UTR) cultured at the indicated temperatures. The box indicates the representative bands that are shown as inserts in Fig 1D. Bands seen under the main band in each lane appear to have the same relative density as Gfp and are consistent with the presence of Gfp degradation products in these samples. (TIF) [file pone.0214521.s001.tif]

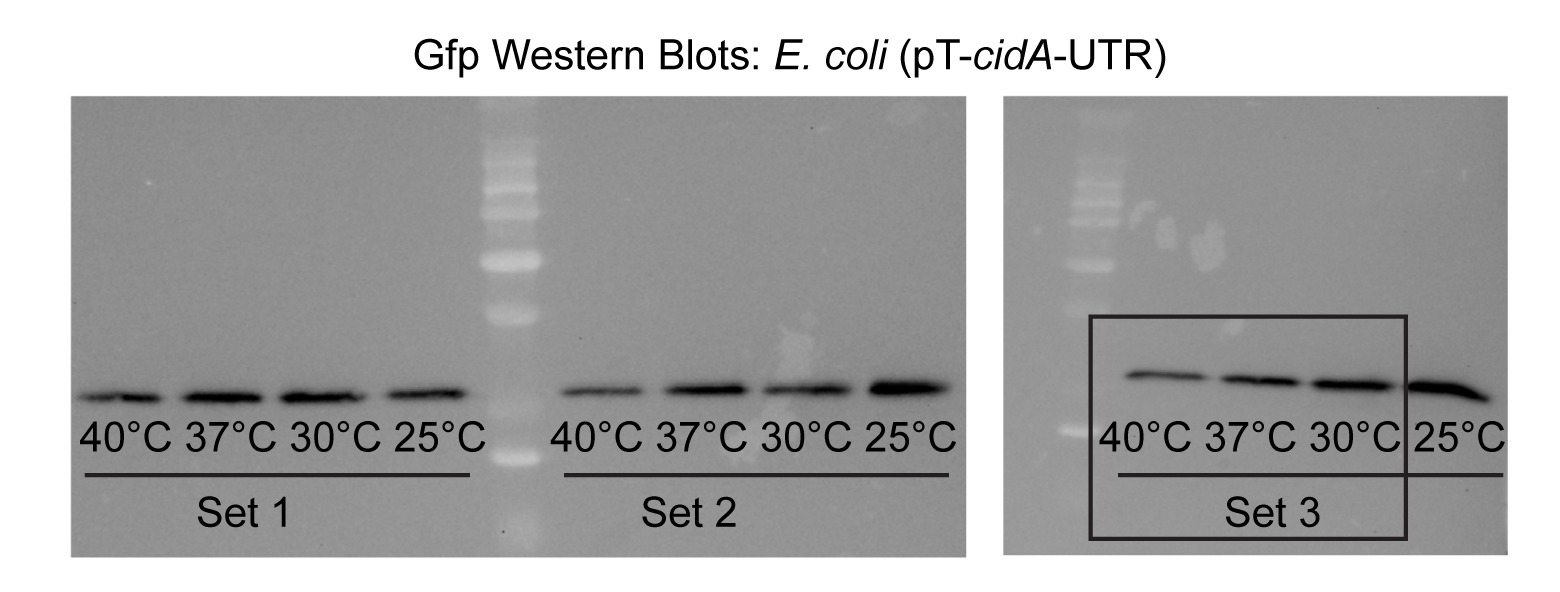

Supplement: S2 Fig — Two gels containing Gfp western blots performed in triplicate with E. coli (pTcidA-UTR) cultured at the indicated temperatures. Data resulting from growth of the reporter strain at 30°C, 37°C and 40°C were selected for inclusion in the presented study. The box indicates the representative bands that are shown as inserts in Fig 2C. (TIF) [file pone.0214521.s002.tif]

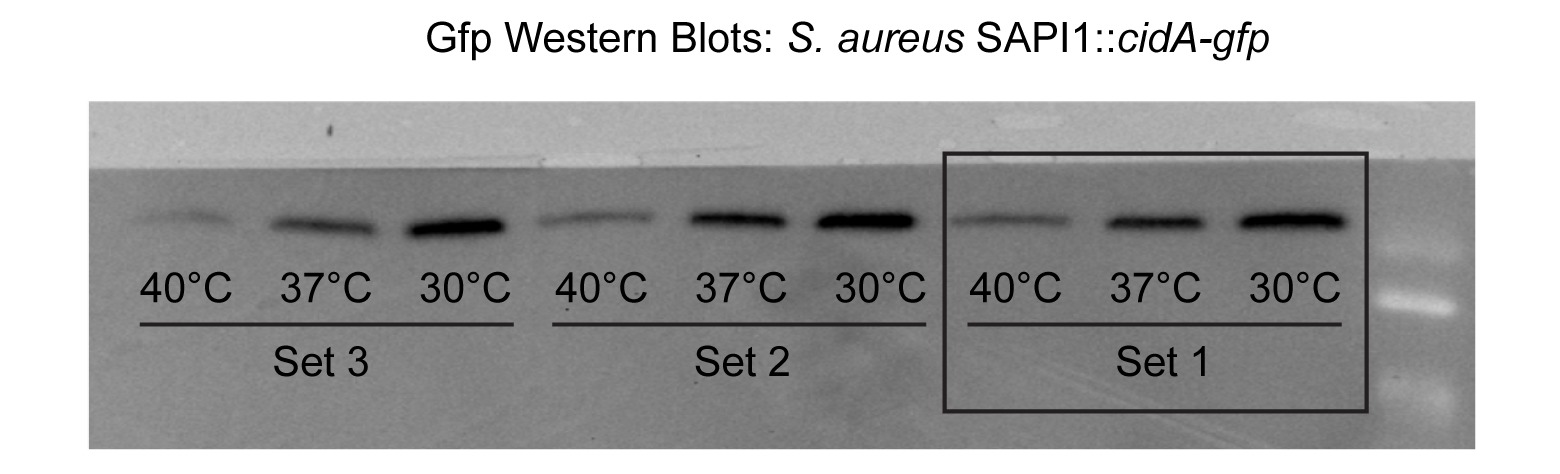

Supplement: S3 Fig — Whole gel image of triplicate Gfp western blot using S. aureus SAPI1::cidA-gfp cultured at the indicated temperatures. The box indicates the representative bands that are shown as inserts in Fig 6B. This blot was cut prior to exposure to the primary antibody in order to eliminate antibody absorption by Protein A. (TIF) [file pone.0214521.s003.tif]

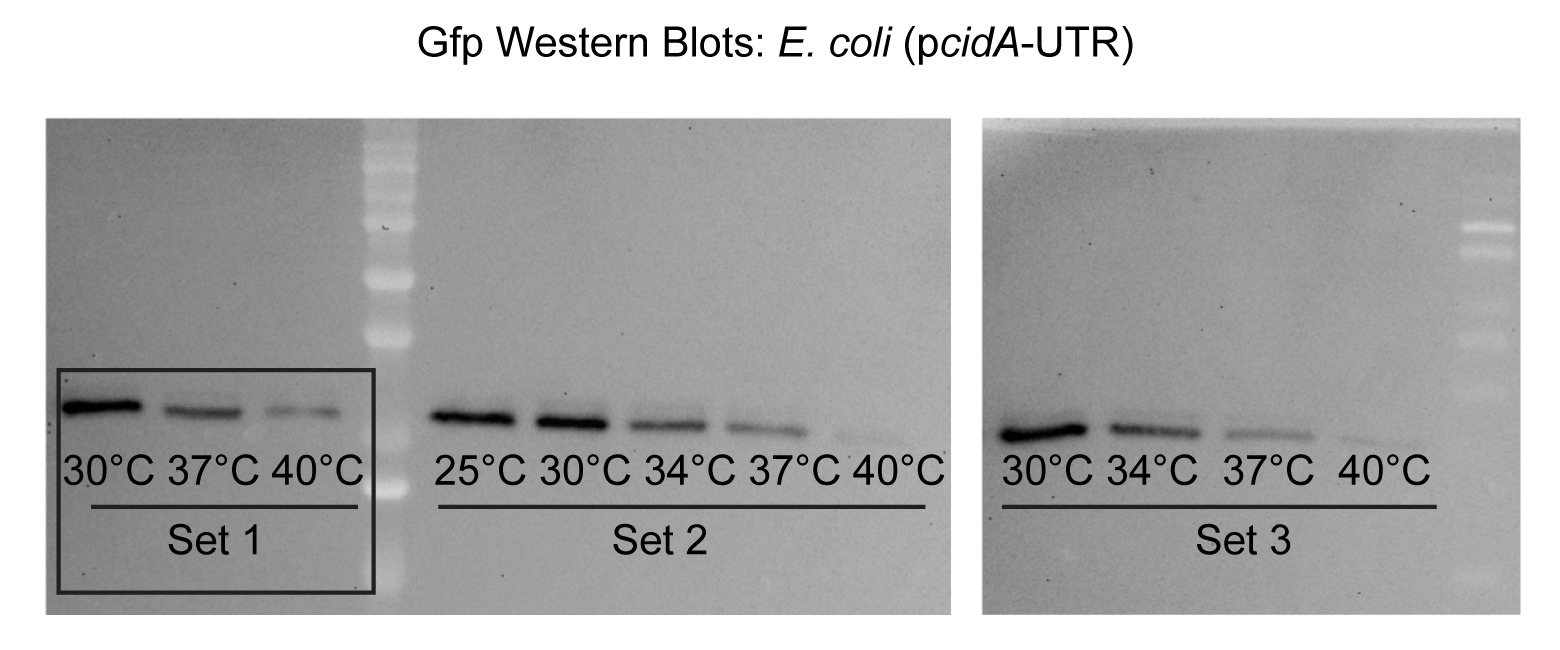

Supplement: S4 Fig — Two gels containing Gfp western blots performed in triplicate with E. coli (pcidA-UTR) cultured at the indicated temperatures. Data resulting from growth of the reporter strain at 30°C, 37°C and 40°C were selected for inclusion in the presented study. The box indicates the representative bands that are shown as inserts in Fig 3C. (TIF) [file pone.0214521.s004.tif]

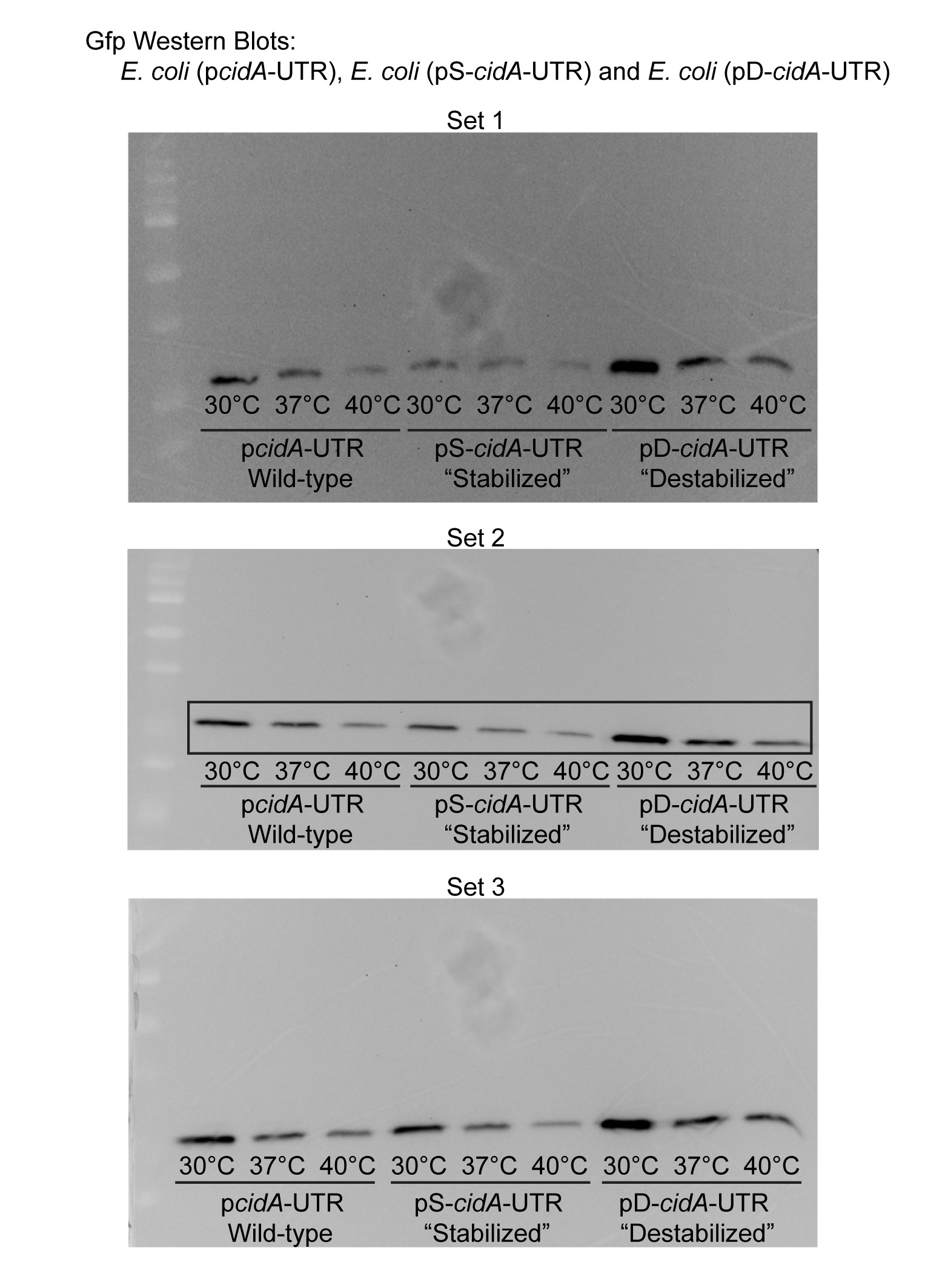

Supplement: S5 Fig — Three gels containing Gfp western blots performed in triplicate with E. coli (pcidA-UTR), E. coli (pS-cidA-UTR) and E. coli (pD-cidA-UTR) cultured at the indicated temperatures. The box indicates the representative bands that are shown as inserts in Figs 4C and 5C. (TIF) [file pone.0214521.s005.tif]
